# Targeting Neuroinflammation to Alleviate Chronic Olfactory Dysfunction in Long COVID: A Role for Investigating Disease-Modifying Therapy (DMT)?

**DOI:** 10.3390/life13010226

**Published:** 2023-01-13

**Authors:** Arianna Di Stadio, Evanthia Bernitsas, Ignazio La Mantia, Michael J. Brenner, Massimo Ralli, Luigi Angelo Vaira, Andrea Colizza, Carlo Cavaliere, Matteo Laudani, Teresa C. Frohman, Marco De Vincentiis, Elliot M. Frohman, Marta Altieri

**Affiliations:** 1 GF Ingrassia Department, Otolaryngology, University of Catania, 95124 Catania, Italy; 2 Multiple Sclerosis Center, Neurology Department, Wayne State University, Detroit, MI 48202, USA; 3 Otolaryngology Department, Michigan University, Ann Arbor, MI 48109, USA; 4 Department of Sense Organs, La Sapienza University, 00185 Rome, Italy; 5 Oro-Maxillo-Facial Department, University of Sassari, 07100 Sassari, Italy; 6 Department of Neurology, University La Sapienza, 00185 Rome, Italy; 7 Distinguished Senior Fellows (Sabbatical), Laboratory of Neuroimmunology of Professor Lawrence Steinman, Stanford University School of Medicine, Stanford, CA 94305, USA

**Keywords:** COVID-19, anosmia, hyposmia, smell disorders, multiple sclerosis, disease-modifying therapy, TDM, olfactory, neuroinflammation, SARS-CoV-2, olfactory training, long COVID, long-haul COVID, post-acute sequelae of SARS-CoV-2 infection, PASC, interferon, threshold detection identification, smell, olfaction

## Abstract

Chronic olfactory dysfunction after SARS-CoV-2 infection occurs in approximately 10% of patients with COVID-19-induced anosmia, and it is a growing public health concern. A regimen of olfactory training and anti-neuroinflammatory therapy with co-ultramicronized palmitoylethanolamide with luteolin (um-PEA-LUT) has shown promising results in clinical trials; however, approximately 15% of treated patients do not achieve full recovery of a normal olfactory threshold, and almost 5% have no recovery. Disease-modifying therapies (DMTs), which are used to treat autoimmune neuroinflammation in multiple sclerosis (MS), have not been studied for treating persistent inflammation in refractory post-COVID-19 smell disorder. This study evaluated COVID-19-related smell loss and MS-related smell loss, comparing the responses to different therapies. Forty patients with MS and 45 reporting post-COVID-19 olfactory disorders were included in the study. All patients underwent nasal endoscopy and were evaluated by using validated Sniffin’ Sticks testing. The patients with long COVID were treated for three months with um-PEA-LUT plus olfactory training. The patients with MS were treated with DMTs. Olfactory functions before and after treatment were analyzed in both groups. At the experimental endpoint, 13 patients in the COVID-19 group treated with um-PEA-LUT had residual olfactory impairment versus 10 patients in the MS group treated with DMTs. The severity of the persistent olfactory loss was lower in the MS group, and the patients with MS treated with IFN-beta and glatiramer acetate had the preservation of olfactory function. These data provide a rationale for considering prospective trials investigating the efficacy of DMTs for post-COVID-19 olfactory disorders that are refractory to um-PEA-LUT with olfactory training. This study is the first to consider the role of DMT in treating refractory post-viral olfactory loss in patients with long COVID.

## 1. Introduction

Post-COVID-19 chronic olfactory dysfunction is associated with a diminished quality of life, safety risks, and impaired social well-being. Individuals with olfactory deficits report anxiety about personal hygiene; increased rates of depression; a reduced enjoyment of food; and safety concerns due to their inability to detect smoke, gas, and toxins. Olfactory dysfunction is thought to have a neuroinflammatory mechanism, and it is an early manifestation of neurodegenerative central nervous system (CNS) diseases, such as multiple sclerosis (MS), Parkinson’s disease (PD), Alzheimer’s disease (AD), and Lewy body disease (LBD) [1]. These conditions share a similar etiopathogenesis with neuroinflammatory and neurodegenerative components. The initial loss of olfactory function in early-stage disorders is related to the neuroinflammatory phase, and only later does it progress to neurodegeneration [1]. Neuroinflammatory processes are more easily treatable than neurodegenerative processes because inflamed neuronal tissue can recover from injury and regenerate, in addition to the mainly anti-inflammatory effect of FDA-approved medications; this regenerative capacity is reduced or lost with neurodegeneration.

Neuroinflammation contributes to the pathogenesis of MS and post-viral olfactory loss after COVID-19 [2], which may explain the shared predisposition to olfactory dysfunction in both disorders [1,2]. MS is treated using immune-modulating drugs that suppress neuroinflammation to reduce the risk of disease progression [3,4]. The incidence of smell alterations in MS ranges from 30 to 75% [5,6,7,8,9,10,11], and the olfactory deficit is correlated with disease duration and Expanded Disability Status Scale (EDSS) scores. Disability also correlates with reduced olfactory threshold, detection, and identification (TDI) scores [12]. Olfactory function is often more severely impaired in the early stage of MS versus later in the disease, and this temporal relationship suggests that neuroinflammation, rather than neurodegeneration, is primarily responsible for smell alterations [12]. In the very early phases of MS, patients suffer from smell threshold reduction, and with disease progression, their identification capacity becomes more affected [13,14].

In MS, inflammation, demyelination, and neurodegeneration take place, but the patterns and sequences vary [4,15]. The most common type of MS is relapsing-remitting MS (RRMS), in which neuroinflammation leads to injury to the myelin sheath, and neurodegenerative changes are limited; then, after a variable time, RRMS often transitions to the secondary progressive form of MS, in which neurodegeneration predominates. Neuroinflammation activates the central nervous system (CNS) immune system [15] and induces the production of inflammatory cytokines that polarize microglia to the M1 (pro-inflammatory) phenotype [16]. Disease-modifying therapies (DMTs) modulate the immune response to reduce the frequency and severity of relapses and to delay the onset of secondary progressive MS [17].

Neuroinflammation has been implicated in olfactory dysfunction in patients with COVID-19; severe acute respiratory syndrome coronavirus 2 (SARS-CoV-2) infection causes direct injury and inflammatory changes [18,19]. The virus induces inflammation of the olfactory epithelium [18] and then spreads to the olfactory bulbs and superior brain centers [20], resulting in persistent olfactory loss [21]. The acute neuroinflammation that causes long COVID [18] can be treated by anti-neuroinflammatory drugs [21,22,23,24]. Various studies have successfully used oral supplements containing ultramicronized palmitoylethanolamide and luteolin (um-PEA-LUT) [21,22,23,24] to counteract neuroinflammation and to treat the olfactory disorders related to long COVID. In a previous study, despite approximately half of patients recovering normal olfactory function, nearly 30% had incomplete recovery, and 8% did not recover at all [22]. Therefore, additional treatments are needed to treat those patients who do not benefit from um-PEA-LUT.

The therapeutic options for persistent post-viral olfactory loss are limited, and novel approaches are needed. Regimens combining um-PEA-LUT therapy with olfactory training have emerged as promising first-line therapies, but these treatments do not restore olfaction for all affected individuals. The advantages of um-PEA-LUT are (i) its favorable safety profile, with the absence of toxicity and adverse effects; (ii) its favorable bioavailability, with a minimal risk of overdose; (iii) its well-tolerated treatment, even in the presence of swallowing disorders; (iv) its efficacy in treating some olfactory loss, by reducing neuroinflammation and neurodegeneration; and (v) its relatively low cost and availability. The disadvantages of um-PEA-LUT include various responses to therapy, differences in pharmacokinetics (variable absorption or metabolism), and difficulty in predicting responses to therapy.

We hypothesized that, whereas um-PEA-LUT would reverse COVID-19 olfactory loss, DMT would result in the stabilization of olfactory function in patients with MS. The findings of the analyses can inform investigations into refractory post-COVID-19 olfactory loss (e.g., hyposmia not responding to PEA-LUT), including testing whether these patients might respond to DMT. The use of DMT to treat COVID-19 has previously been proposed based on the ability of DMT to modulate neuroinflammation [25], but olfactory outcomes have not been assessed. In this study, we compared the olfactory function of patients with MS treated with DMT to the olfactory function of patients with long COVID treated with PEA-LUT. This comparison affords insights into the differences between DMTs in preserving olfactory function, the patterns of olfactory recovery with MS versus olfactory recovery post-COVID-19, and hypothesis generation regarding a potential role for investigating DMTs as therapeutics for olfactory dysfunction in long COVID.

Incidentally, this study is not designed to test the hypothesis that DMT might have efficacy in COVID-19 anosmia (or long COVID). In that respect, this study is mainly a hypothesis-generating work based on the expectation that DMT will stabilize olfactory function in MS, whereas PEA-LUT will promote recovery from COVID-19 anosmia.

## 2. Materials and Methods

This prospective study was conducted from April 2021 to December 2021 at the Policlinico Umberto I University Hospital of Rome and at the University Hospital of Sassari. All patients included in the study signed a written informed consent form to participate, including an agreement regarding the publication of their data for research purposes.

Patients affected by MS were recruited in the Neurological Department of Policlinico Umberto I, while patients with smell alteration caused by COVID-19 (long COVID) were recruited in the Head and Neck Department of Sassari University Hospital.

This study was approved by the Internal Review Board (IRB) of La Sapienza University of Rome with number 6941. No randomization was performed. All patients were tested at baseline (T0) and three months after treatment.

Both centers, supervised by the same research coordinator (A.D.S.), performed the same screening for olfactory disorders, which consisted of an endoscopic examination of the nasal cavities and an evaluation of smell thresholds (T), detection (D), and identification (I) by using Sniffin’ Sticks as previously described [22,23]. The patients with MS underwent a neurological examination and an evaluation of disability with the Expanded Disability Status Scale (EDSS).

***Inclusion criteria:*** Patients with RRMS or persistent olfactory loss after COVID-19.

**MS:** All outpatients affected by MS for at least three years and under treatment with any DMTs; negative for COVID-19, as confirmed by a nasopharyngeal swab; and with no known COVID-19 infection in the last two years.

**Long COVID:** All outpatients aged 18 to 60 years with a confirmed history of COVID-19 (a nasopharyngeal swab positive for SARS-CoV-2) and anosmia/hyposmia persisting ≥ 180 days after a negative COVID-19 nasopharyngeal swab.

***Exclusion criteria***

All patients: The following exclusion criteria applied to all potential patients in both groups: impaired cognitive function, medical therapy with medications that have possible effects on olfactory function, the presence of rhinological disorders (sinusitis, rhinosinusitis, sinonasal polyposis, atrophic rhinitis, or allergies), a history of chemoradiotherapy of the head and neck region, a history of stroke or neurotrauma, severe nasal blockage from stenosis of deformity, a severe psychiatric illness (e.g., schizophrenia, bipolar disorder, and olfactory hallucination), or previous sinonasal or nasopharyngeal tumors.

**Patients with MS**: Patients not on DMT.

**Long COVID**: Patients referred for COVID-19 infection without confirmed swab.

The following ***demographic data*** were collected:

**Long COVID:** sex, age, severe diseases, tobacco/alcohol use, and time elapsed since negative COVID-19 test.

**MS:** sex, age, age at diagnosis, tobacco/alcohol use, educational level, type of MS (RRMS, clinically isolated syndrome (CIS), primary progressive MS (PPMS), or secondary progressive MS (SPMS), DMT used, EDSS, relapse within 6 months prior to the enrollment in the study, the availability of MRI, and the presence of new lesions at study enrollment.

**Modality of the treatment**

The patients with MS were asked about their DMT therapy and standard olfactory training only when they were identified as having an olfactory deficit.

The patients in the COVID-19 group were treated using um-PEA-LUT (palmitoylethanolamide 700 mg + luteolin 70 mg) (Glialia ^®^, Epitech group, Saccolongo (PD) Italy) plus olfactory training (OT). The OT protocol was performed using organic essences of lemon, rose, cloves, and eucalyptus. To perform OT, the patients were asked to sniff an essence for 5–10 s and then to wait 40 s before sniffing a new one. This protocol has been shown to be effective for COVID-19 smell loss in several prior studies [22,23,24].

Because several studies [22,23,24] have shown the superiority of um-PEA-LUT compared to olfactory training alone for the treatment of smell loss due to COVID-19, we decided not to include a control COVID-19 group that withheld um-PEA-LUT. Two recent clinical trials [20,21] and a longitudinal study [22] compared the treatments “um-PEA-LUT plus olfactory training” and “olfactory training alone”; in all studies, the combination therapy was better than the control therapy (olfactory training alone), based on data from over 300 patients [22,23,24]. The combination of um-PEA-LUT and OT is noted in a 2022 Cochrane review on treatments for smell disorders caused by SARS-CoV-2 infection [26].

### 2.1. Assessment of Olfactory Dysfunction

The Sniffin’ Sticks battery test was administered by following a previously established protocol [22,23] and using pen-like devices filled with odorants. The clinicians conducting the tests were blinded to the treatment groups of the patients. Three score subtests were conducted to measure olfactory function: (1) detection threshold (“T”, the lowest concentration at which an odor can be perceived); (2) odor discrimination (“D”, the ability to distinguish between odors); and (3) odor identification (“I”, the ability to assign names to odors).

The possible scores ranged from 1 to 16 for the detection threshold subtest and from 0 to 16 for both the discrimination and identification subtests. A TDI “sniff score” was then calculated for each patient. Anosmia was defined with a score of <17, hyposmia was defined with a score of 17 to 30.75, and normosmia was defined with a score of ≥31.

In the first test, the odor detection threshold was determined using a three-option, yes/no staircase, and forced-choice procedure with the odorant n-butanol. The participants were presented with triplets of odorant pens and asked to identify the pen containing n-butanol when presented with two blank distractor pens. In the second subtest, odor discrimination ability was assessed using 16 triplets of odorants: within each triplet, two pens contained the same odorant, while the third pen contained a different odorant. In the forced-choice procedure, the participants were asked to detect the odd pen in each triplet. During the odor identification task, the participants were presented with 16 common odors. Using a multiple-choice answering format, they were asked to select which of the 4 odor labels matched the presented odor.

The assessment of olfactory function was performed at baseline (T0) and three months after treatment (T1) exactly as previously described [23].

### 2.2. Analyses of Data and Statistical Tests

Both within and between analyses were performed. The results of the olfactory tests of the patients with MS and the patients with long COVID (pre- and post-treatment) were compared using one-way ANOVA; T, I, and D were compared individually between the two groups before and after treatment (COVID-19). Then, the total olfactory scores (TDI) were compared between the MS (T0 and T1) and long COVID (T0 and T1) groups by using a two-tailed t-test. In both groups, the differences between genders were compared. In the MS group, Pearson correlations were calculated to assess the correlations between EDSS and T, I, D, or TDI. Odds ratios were calculated for the MS group to compare patients with smell alteration affected by different forms of the disease. In the presence of smell disorders, chi-square was performed to analyze the differences in the outcomes between DMTs. In both the MS and COVID-19 groups, odds ratios were calculated to compare the olfactory function of women and men. Finally, we calculated the odds ratios for smokers in both the MS and COVID-19 groups. Statistical significance (*p*) was set as <0.05. All tests were performed by using Stata^®^.

## 3. Results

Table 1 summarizes the characteristics of the groups.

### 3.1. Within-Group Comparison

#### 3.1.1. Multiple Sclerosis Group

Eight patients were excluded following the exclusion criteria. A total of 40 patients were included (28 women and 12 men). The average age was 48.5 ± 13.7 years. In total, 29 patients (61.7%) were affected by RRMS, 8 (17%) suffered from SPMS, and 3 (6.5%) had PPMS. Furthermore, 20 patients were smokers (11 women and 9 men). The average age at diagnosis was 32.8 ± 10.7 years (95% CI: 14–58).

In the MS group, the EDSS median score was 2.1 (range 0–7; SD: 2.1). Four patients (three women and one man) with RR and a woman with SP had a relapse with the presence of one or more contrast-enhancing lesions related to MS. Only five patients (four with RR and one with SP) presented with a new non-enhancing lesion when comparing magnetic resonance imaging (MRI) performed at the recruitment time with that performed 12 months prior.

**Figure 1:**
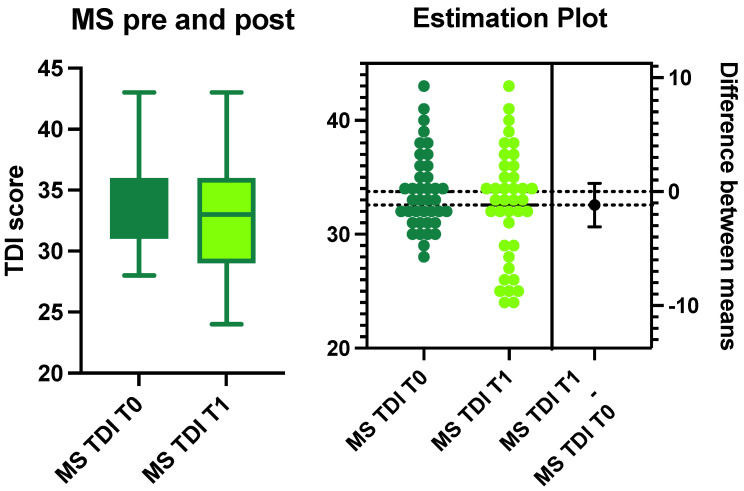
The graph shows the difference in composite olfactory threshold, discrimination, and identification (TDI) scores for patients with multiple sclerosis (MS) at T0 and T1. No statistically significant variances were observed between T0 and T1 in these patients, as shown in the estimation plot (right side of the image).

The average TDI score in the entire sample was 33.5 ± 4.9 (normal olfactory function) at T0. The average scores of T, D, and I were 7.7 ± 2.5, 11 ± 2.6, and 13.4 ± 1.4, respectively. Only 25% of the sample (10 patients, 6 women and 4 men) suffered from olfactory impairment at T1. All were considered hyposmic based on their TDI score (Figure 1).

The average TDI score of the patients affected by smell disorders at T1 was 26.4 ± 1.7. Looking specifically at the T, D, and I scores, the average and SD values were 4.9 ± 6.9, 8.3 ± 2, and 12.4 ± 1.3, respectively (Figure 1). No statistically significant differences were identified in the TDI scores of the patients with MS before and after treatment (ANOVA: *p* > 0.05) (Figure 1).

When analyzing olfactory function in relation to the forms of MS, we observed that 81.8% of the patients were affected by RR (nine patients—four men and five women); the remaining two women (18.2%) suffered from non-active SP. The patients with RR did not have a significantly increased risk of suffering from smell disorders compared to those with SP (*p* = 0.7).

Analyzing the DMT therapeutics administered and their impact on olfactory alterations, we identified statistically significant differences in the patients who were treated with IFN-beta 1a and glatiramer acetate (GA) when compared with patients who were treated with Fingolimod, Dimethylfumarate, Natalizumab, and Teriflunomide (χ: *p* = 0.02). None of the patients on IFN-beta 1a or GA had olfactory dysfunction. No statistically significant differences were identified when comparing Fingolimod, Dimethyl-fumarate, Natalizumab, and Teriflunomide (χ: *p* = 0.8). (Table 2).

The EDSS score was inversely correlated with the identification score (Pearson, *p* = 0.006). No other statistically significant correlations were identified between the EDSS score and the other olfactory scores. The risk of suffering from a smell disorder was higher (odds ratio: 1.4) for men than for women, without a statistically significant value (*p* = 0.5).

#### 3.1.2. Long COVID Group

In the long COVID group, 45 (31 women and 14 men) patients were recruited, with an average age of 39.5 ± 12.8. Twelve were smokers (six women and six men). In this group, the following co-morbidities were identified: thyroid disease (three patients), hypertension/cardiovascular disease (four patients), and a history of tumors (two patients). Twenty-two patients had no comorbidities and were non-smokers.

**Figure 2:**
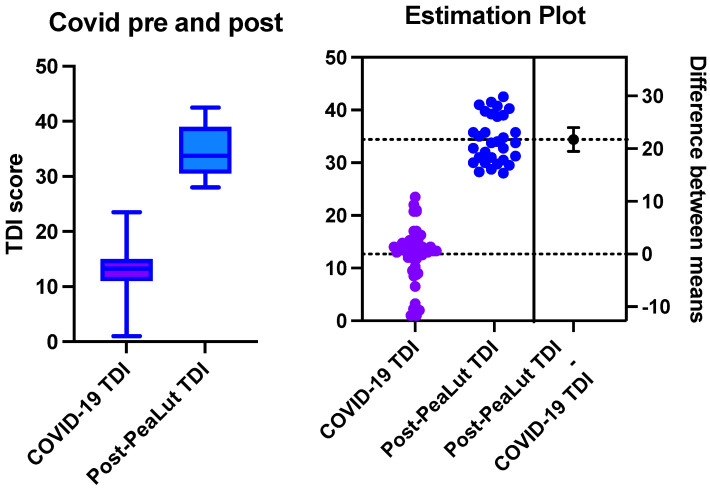
The graph shows the differences in composite olfactory threshold, discrimination, and identification (TDI) scores of patients with COVID-19 (MS) at T0 and T1. Statistically significant variances were observed between T0 and T1 in these patients. as shown in the estimation plot (right side of the image).

None of the patients had normal olfactory scores at T0; 2 were hyposmic, and 43 were anosmic. The average TDI score was 12.6 ± 5.1 (Figure 2). Looking specifically at the threshold (T), detection (D), and identification (I) scores, the average and standard deviations were 3.8 ± 1.8, 4.6 ± 2, and 4.1 ± 2.1, respectively.

Women were at a higher risk of suffering from smell disorders than men (odds ratio: 4.9; *p* = 0.0005).

After 3 months of treatment with um-PEA-LUT (T1), 71.2% of the patients (32 subjects) recovered normal olfactory function or borderline normal (defined as a TDI score of 28.5 or more but less than 30) (Figure 2). Comparing pre- and post-TDI scores, we observed significant differences in these patients (*p* < 0.0001) (Figure 2).

**Figure 3:**
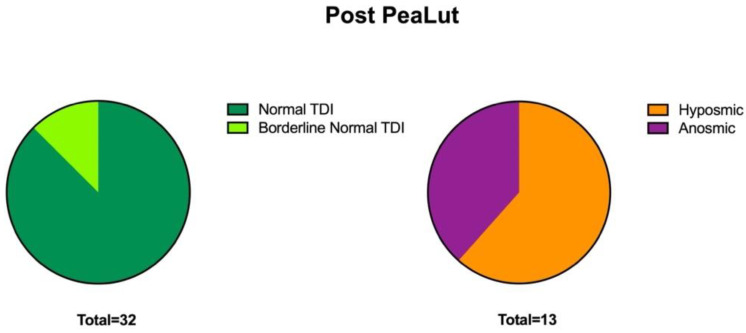
The figure shows the details of the recovery of the smell function in patients with COVID-19 after being treated with PEA-LUT and olfactory rehabilitation for 3 months.

The average TDI score was 34.3 ± 4.6 (CI 95%: 28.5–41.5). The 28.8% of patients (13 patients) who did not recover showed an average TDI score of 18.5 ± 8.1 (CI 95%: 9–27.25) (Figure 2 and Figure 3).

### 3.2. Between-Group Comparison

Patients with Multiple Sclerosis versus Patients with COVID-19

Figure 2 and Figure 3 show a comparison of the T, D, I, and TDI scores between the MS and COVID-19 groups before and after PEA-LUT treatment. Statistically significant differences were identified (ANOVA: *p* = 0.0001) between the MS and COVID-19 groups pretreatment. The differences were statistically significant for T (*p* < 0.0001), D (*p* < 0.0001), I (*p* < 0.0001), and composite TDI (*p* < 0.0001) scores when comparing the MS group to the COVID-19 group pretreatment.

**Figure 4:**
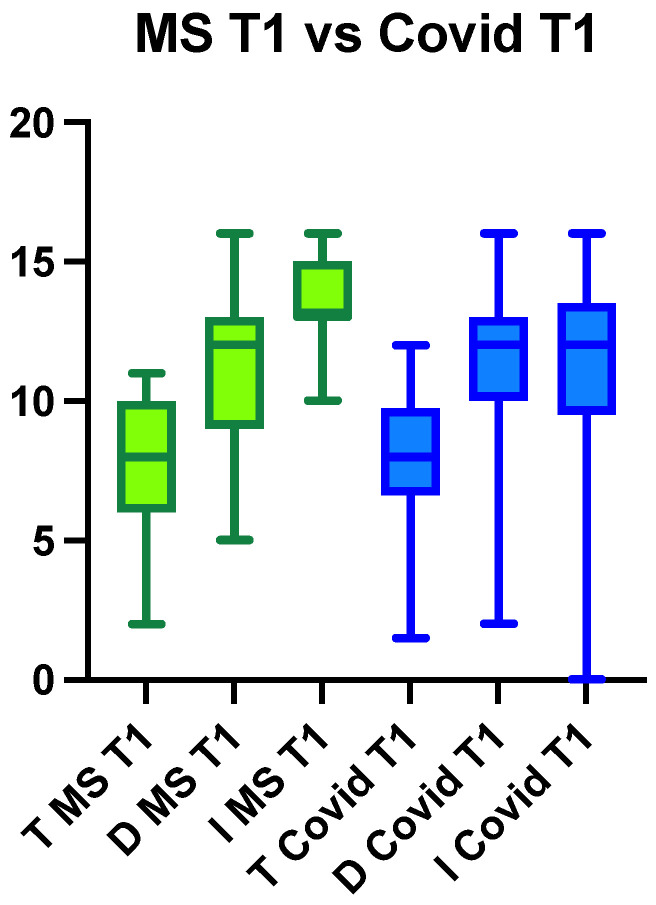
The graph shows the detailed differences in threshold, detection, and identification (TDI Sniffin’ Score) between patients with MS at T1 and patients with COVID-19 after treatment.

After treatment, no statistically significant differences were observed between the patients with MS and patients with COVID-19 (Figure 4).

The analysis of smokers showed that patients with MS had an increased risk (5.1 odds ratio, *p* = 0.02) of suffering from smell alterations compared to patients with COVID-19.

## 4. Discussion

In this study, we compared patients with COVID-19-induced olfactory loss before and after treatment to patients with MS undergoing treatment with DMTs. As predicted, DMTs were associated with the protection/preservation of olfactory function in MS. The patients with olfactory impairment after COVID-19 had various benefits from um-PEA-LUT, with non-responders potentially having either advanced neuroinflammation or permanent damage/neurodegeneration that precluded recovery. The patients with MS treated with IFN-beta 1a and GA had improved olfactory function when compared to the patients undergoing treatment with other DMTs; however, given the small number of patients in each DMT group, the results should be considered preliminary.

The patients with smell disorders caused by SARS-CoV-2 infection had baseline TDI scores that were substantially lower than the TDI scores of the patients with MS. We hypothesized that DMTs would prevent progressive olfactory loss (reduce neuroinflammation); so, the stable olfactory function at T0 supports this theory. In contrast, when analyzing the effect of um-PEA-LUT, reduced neuroinflammation was expected to restore function, which was also corroborated by the findings.

SARS-CoV-2 injures the sensory neuroepithelium, olfactory bulb, and higher centers due to the neuroinflammation induced by the virus [18,27,28], and um-PEA-LUT restored olfactory function in 71.2% of the participants when used for three months. In COVID-19 olfactory dysfunction, neuroinflammation has a brisk onset after infection, making it a compelling candidate for treatments targeting inflammatory mechanisms.

One-quarter of the patients with MS who presented with smell alterations had mild severity olfactory loss (an average TDI score of 26.4), which was inversely correlated with their EDSS scores. This observation agrees with a prior study that found smell alterations in the early inflammatory phase of MS [12]. In our study, the patients mainly had a deficit in odor identification. In prior studies, impaired identification was linked to the neurodegeneration of the CNS, which may occur as a sequela of neuroinflammation [13,14]. In the present study, the impaired identification of olfactory stimuli was correlated with a low EDSS score and a neuroinflammatory state.

Compared with patients with long COVID, smokers with MS had a higher risk of being affected by smell disorders (odds ratio: 5.1, *p* = 0.02). This observation might link alterations in odor identification to a higher susceptibility to the damage of the nasal neuroepithelium rather than to central neurodegeneration [29]. The peripheral component of the olfactory pathways seems to be more sensitive to chemical damage in patients with MS than in patients with COVID-19 [28]; in addition, smoking has a negative effect on neuroinflammation, which exposes patients with MS with a smoking history to a higher risk of disease progression [30]. Another hypothesis regarding the pathogenesis of impaired odor identification involves alterations in memory [31] or the psychological state, which impedes hedonic perception [32,33].

Most of the patients with MS in our study had preserved olfactory function when using DMTs, suggesting that the anti-inflammatory mechanism of action of DMTs could be protective for olfaction. None of the patients treated with IFN-B1a or GA suffered from olfactory loss. This favorable outcome could be attributed to (i) a mild form of MS in these patients; (ii) the absence of inflammation affecting the olfactory pathway; or (iii) the beneficial effect of these two treatments on olfactory function. Further investigation is necessary to elucidate the effects of IFN-B1 and GA on olfactory function in the context of MS. The putative benefits in the setting of MS also suggest a potential role for investigating DMTs in patients with COVID-19 olfactory loss.

When we compared the patients with MS and the patients with long COVID, we observed that COVID-19 caused a more severe olfactory deficit than MS, consistent with the massive inflammation caused by the virus. Pretreatment, the patients with long COVID had notably lower TDI scores (severe hyposmia to anosmia) than the patients with MS. In contrast, after three months of treatment with PEA-LUT, the patients who recovered olfactory function had higher TDI scores than their MS counterparts. Because the olfactory thresholds seem to represent the active neuroinflammation phases [12,13,14,15], um-PEA-LUT could modulate the neuroinflammatory process, as shown by the recovery of olfactory function. Unfortunately, 28.8% of the patients treated with um-PEA-LUT did not recover normal olfactory capacities. An incomplete recovery may reflect the different phases of neuroinflammation [34], the variable genetics of the host [35], or differences in the severity of injury (e.g., more severe neuroinflammation refractory to the treatment).

We observed that the patients with MS who were treated with IFN-beta 1a and GA had preserved olfactory capacity, consistent with the control of neuroinflammation, but the mechanism has not been investigated with biomarkers. To date, experimental studies have suggested that both IFN-beta 1a and GA can modulate neuroinflammation and reduce pro-inflammatory cytokines. IFN-beta 1a increases IL-10 (anti-neuroinflammatory), acting indirectly on the microglia state [36]; IL-10 might induce a favorable microglial phenotype, reducing neuroinflammation and facilitating the recovery of olfactory function, as has been shown for memory [37]. GA also has anti-inflammatory properties [38] and can improve blood perfusion by acting on platelet aggregation [39].

In COVID-19, there are a least two possible mechanisms responsible for neuroinflammation. One of these mechanisms is the inflammatory process, which is driven by the virus, and the other is the increase in platelet aggregation, which reduces blood perfusion, inducing ischemic stress and worsening the neuroinflammation [39,40]. Future work is needed to explore whether IFN-1B or GA might ameliorate olfactory function when um-PEA-LUT treatment is ineffective. Combination therapy might also be a future area of research, as PEA might reduce the adverse effects of IFN-beta 1a [41], which is the most important limitation of using this drug. PEA can reinforce the anti-inflammatory capacity of GA, further improving the efficacy of combined treatment [38]. Luteolin (LUT) is a natural bioflavonoid that modulates the immune response, and it can be protective in cases where IFN-B 1a or GA induces allergic reactions [42]; in addition, um-PEA-LUT modulates mastocyte activation, reducing the risk of excessive flu-like symptoms when using IFN-beta 1a and GA [43,44].

Although this study employed a complex experimental design, the common theme relates to reducing neuroinflammation in order to prevent functional loss or promote the recovery of olfactory function. We studied groups of patients with very different diseases, MS and COVID-19, but both involve neuroinflammatory mechanisms. Although the origins of neuroinflammation are extremely different—immune-mediated for MS and virus-induced for COVID-19—the underlying mechanisms that sustain the inflammation are similar. Microglia and pro-inflammatory cytokines drive the loss of olfactory function. The analysis of functional recovery in COVID-19 and functional preservation in MS affords indirect clinical evidence of the ability to modulate neuroinflammation.

### Limitations

The main limitation of this study is that the comparisons of um-PEA-LUT versus DMTs were conducted in parallel populations with different underlying disorders. Therefore, differences in treatment outcomes may reflect underlying differences in disease profiles rather than differences in the efficacy of the therapeutics administered. As a result, the use of IFN and GA in COVID-19 requires further investigation. Future prospective randomized trials are necessary to evaluate whether DMTs can be safely and efficaciously applied to long COVID or other forms of post-viral olfactory loss. The other limitations of this study are that we did not measure inflammatory biomarkers in any of the groups. So, the role of reducing inflammation in olfactory function is speculative; clinical findings only allow an inference regarding the mechanism of effect. The sample size, despite the generally balanced groups, is quite small. MS and olfactory dysfunctions in post-COVID-19 are conditions that affect more women than men [18], meaning that the lack of balance is most likely related to the difference in baseline prevalence. Additional limitations are related to the differences in the age range of the study subjects with MS versus those with COVID-19, potentially confounding comparisons. Finally, the subdivision of patients in the MS group based on the different treatments used led to small sub-groups; this could limit the power of the statistical analyses.

## 5. Conclusions

Neuroinflammation has been implicated in the olfactory impairment observed in both post-viral syndromes and neurodegenerative disorders. Therefore, COVID-19 anosmia and MS, though very different in etiology and clinical course, may share a common anatomic substrate that can be targeted through overlapping therapeutic strategies. um-PEA-LUT is a natural compound with efficacy in treating olfactory impairment due to COVID-19, although a significant number of patients have incomplete recovery from this treatment (which is usually combined with olfactory training). In contrast, DMTs are used in MS to manage neuroinflammation; in this study, the patients treated with either IFN or GA had preserved olfactory function, suggesting protective effects for olfaction. The role of DMTs in treating long COVID is exploratory and undefined. Clinical studies investigating DMT therapies in patients with COVID-19 are needed to determine whether DMT benefits this population.

Combining um-PEA-LUT and DMTs might further reduce neuroinflammation and minimize the adverse effects of treatment. Further work is necessary to define the role of DMT in patients with refractory olfactory loss related to COVID-19, as well as the possible use of um-PEA-LUT in MS or other inflammatory neurodegenerative disorders. Large-scale, prospective studies will play an ongoing role in advancing therapy for these disorders.

## Figures and Tables

**Table 1:** Characteristics of the groups.

	*Age*	*Women, Men*	*Smokers*	*EDSS*	*Comorbidities*
MS	48.5 ± 13.7	28, 12	20	1.9 ± 2.2	n/a
COVID-19	39.5 ± 12.8	31, 14	12	n/a	9 (3 thyroid, 4 cardiovascular, 2 tumor)

**Table 2:** Treatments administered to the patients with multiple sclerosis.

**Treatment**	*Fingolimod (Gilenya)*	*Teriflunomide (Aubagio)*	*Ocrelizumab*	*Cladribine (Mavenclad)*	*Dimethylfumarate (Tecfidera)*	*IFN-beta 1a (Avonex)*	*Natalizumab (Tysabri)*	*IFN-beta 1a (Plegridy)*	*Glatiramer Acetate (Copaxone)*
Number of patients	8	7	4	3	6	1	4	3	2

## Data Availability

The data are available upon reasonable request to the corresponding author.
